# Pediatric Cerebral Venous Sinus Thrombosis: A Case Report

**DOI:** 10.7759/cureus.54302

**Published:** 2024-02-16

**Authors:** Pruthvi Patel, Shirley M Gandhi, Philip P Breton, Tetiana Litvinchuk

**Affiliations:** 1 Internal Medicine, Alabama College of Osteopathic Medicine, Dothan, USA; 2 Internal Medicine-Pediatrics, Alabama College of Osteopathic Medicine, Dothan, USA; 3 Medical School, Alabama College of Osteopathic Medicine, Dothan, USA; 4 Pediatrics, Helen Keller Hospital, Sheffield, USA

**Keywords:** neurology, intraventricular hemorrhage (ivh), pediatric seizure, hydrocephalus, cerebral venous sinus thrombosis (cvst)

## Abstract

Cerebral venous sinus thrombosis (CVST) in infants is a rare vascular disorder that presents with nonspecific symptoms leading to a delay in diagnosis and treatment. Thrombus formation in the cerebral sinuses prevents blood from draining out of the brain leading to local and systemic complications. Here, we present an 11-week-old patient who presented to the emergency department (ED) with three days of lethargy, multiple episodes of projectile emesis, increased fussiness, and downward gaze. A CT scan demonstrated intraventricular hemorrhage (IVH) with acute hydrocephalus. A CT venogram of the cranial vault with contrast showed a large intraluminal thrombus occupying the right transverse sinus and torcula with proximal extension into the left transverse sinus confirming the diagnosis of CVST.

## Introduction

Cerebral venous sinus thrombosis (CVST) occurs when blood clots in the venous sinuses located in the dura mater of the meninges lead to obstruction of blood flow. The obstruction of the sinuses prevents veins from draining the brain leading to decreased cerebrospinal fluid (CSF) absorption and elevated intracranial pressure [1]. As a result, venous infarction and intracranial hemorrhage can occur. The annual incidence of CVST is 8.7 (95%CI, 8.0-9.7) per million [2]. CVST is more common in women than men with a ratio of 3:1 [3]. While it is more common in middle-aged adults, it can present in children in rare cases with an incidence of 6.7 million children each year [4].

The development of venous clots in the body can be attributed to the three pillars marked by Dr. Rudolf Virchow: stasis, hypercoagulability, and endothelial damage [5]. Common risk factors that predispose patients to CVST include pregnancy, oral contraceptives, obesity, head trauma, malignancy, and hematological conditions such as sickle cell disease or blood-clotting disorders which promote a thrombotic state [6]. Children with chronic conditions such as inflammatory bowel disease, systemic lupus erythematosus, and Cushing’s syndrome also appear to be more likely to develop CVST [7,8]. Infectious etiologies can also lead to the development of CVST due to the increase of pro-coagulable factors in an effort to mount an inflammatory response. Studies have shown an association between damage-associated molecular patterns (DAMPs) released by activated leukocytes in the immune response and the formation of thrombi [9].

Symptoms that manifest in a CVST are unique due to neurological involvement and can vary depending on the location of the thrombus. Common clinical manifestations in newborns include seizures, irritability, and extreme sleepiness. Children and adolescents with CVST tend to experience additional symptoms of headache, nausea, vomiting, dizziness, abnormal eye movements, and hemiparesis. If cranial nerves are affected, patients can have tinnitus, facial weakness, deafness, and facial weakness [6]. 

## Case presentation

**Figure 1:**
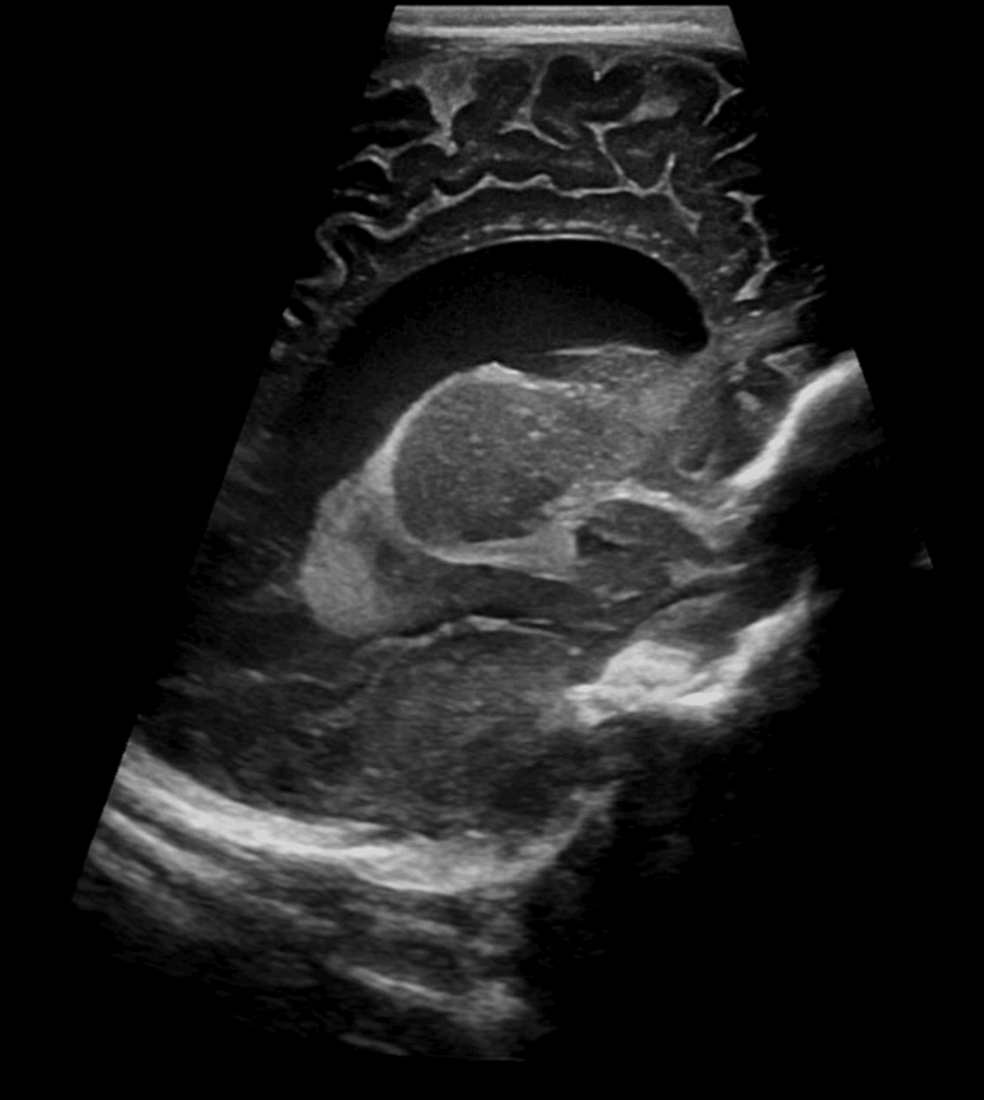
Ultrasound of the head depicting ventriculomegaly.

An 11-week-old infant presented to the emergency department (ED) with three days of lethargy, multiple episodes of projectile emesis, and increased fussiness. When awake, he kept his neck extended and was extremely fussy during supination. He tested positive for respiratory syncytial virus (RSV) a week ago and was placed on azithromycin, but the upper respiratory infection was not associated with any drowsiness or emesis. The patient then had an episode of downward gaze (setting sun eyes) for at least eight hours that resolved on its own. With concerns of seizures, the pediatrician ordered an ultrasound of the head which demonstrated ventriculomegaly and prompted the family to get immediate care (Figure 1).

**Figure 2:**
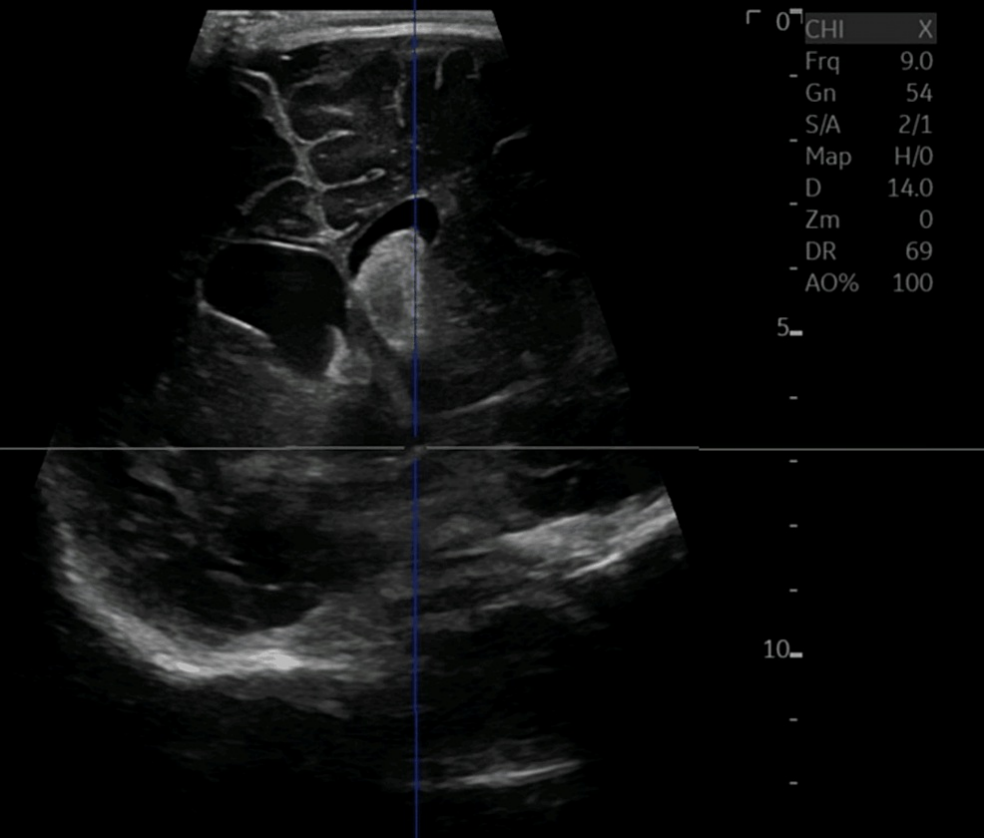
Ultrasound of the head depicting intraventricular hemorrhage.

**Figure 3:**
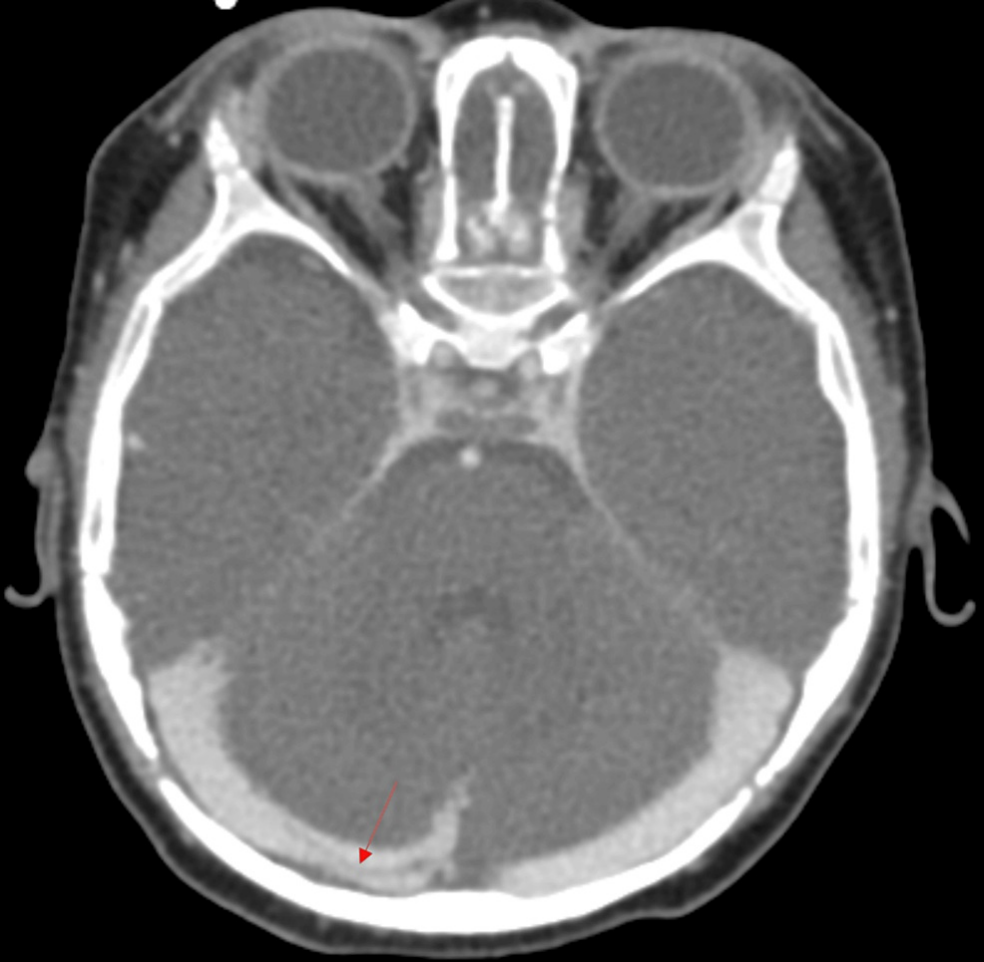
CT venogram of the cranial vault with contrast depicting a resolved thrombus (red arrow) in right transverse sinus.

An ultrasound revealed a moderate to large left intraventricular hemorrhage (IVH) with acute hydrocephalus (Figure 2). The scan showed rounded lateral ventricles, temporal horns, and a rounded third ventricle. These are radiographic signs of hydrocephalus which clinically correlated with his full fontanelle and fussiness. An external ventricular drain (EVD) was placed for symptomatic relief and safely followed by a CT venogram of the cranial vault with contrast. The venogram showed a resolving intraluminal thrombus occupying the right transverse sinus confirming the diagnosis of CVST (Figure 3).

## Discussion

In this case, cerebral venous thrombosis was a contributing factor to the presentation of hydrocephalus. The etiology of the CVST is unknown but previous connections between acute infections and CVSTs have been established [4]. Increased inflammatory markers in acute infections can increase the chances of venous thromboembolism and an upper respiratory infection could lead to a CVST, especially in infants [4,10]. While there was no evidence of RSV meningitis, local and systemic inflammation caused by cytokines and leukocyte migration across the endothelium is thrombogenic. Disruption of vascular integrity is due to increased expression of leukocyte adhesion molecules, complement effectors, VWF, and coagulation factors [10]. The first signs of CVST in this patient were very nonspecific and included lethargy, emesis, and increased fussiness. RSV might not only have caused this CVST but also covered up many presenting symptoms. There was no evidence of hematological disorders, coagulopathies, trauma, or malignancies. Considering this, the most likely etiologies are infectious or cryptogenic. This was followed by a prolonged, eight-hour episode of downward gaze increasing the suspicion for a potential cerebral etiology [11]. Persistent downward gaze is a paralysis of upward gaze that presents in children with hydrocephalus earlier than other physical exam findings such as full fontanelle and enlarged head [11]. The setting sun phenomenon can even occur before it is evident on a CT scan [11]. In anticipation of a potential seizure or meningitis, an ultrasound was ordered, revealing ventriculomegaly.  

Based on imaging and clinical presentation, cerebral etiology became one of the top differential diagnoses. Symptomatic care of the hydrocephalus was managed with an EVD, which helped decrease the intracranial pressure. Then a CT venogram elucidated the CVST in the right transverse sinus. Due to the potentially fatal outcomes of CVST, acute treatment was focused on establishing blood flow in the occluded sinus. The 2019 American Heart Association (AHA)/ American Stroke Association (ASA) statement on the management of stroke in children and neonates recommends antithrombotic therapy with recurrent neurovascular imaging to determine cessation of therapy [12]. Starting anticoagulation using enoxaparin 1 mg/kg subcutaneously every 12 hours for six weeks. His anti-Xa levels were monitored and obtained four hours after his second dose. After two consecutive levels were within the therapeutic range of 0.5-1 units/ml, levels were monitored weekly, then monthly [12,13].  

After addressing the underlying thrombosis, unexpected complications arose when removing the EVD. Physicians realized the sutures used in closing had captured the edge of the drain. Safe removal of the EVD required the patient to be sedated and taken to the operating room. Afterward, his medical regimen was transitioned to Lovenox [14]. The patient later presented with nausea and multiple episodes of emesis daily and developed a subgaleal fluid pocket. A subaponeurotic (subgaleal) fluid collection is an extracranial accumulation of fluid trapped between the scalp aponeurosis and the periosteum [15]. A few weeks later, the patient was brought in due to a CSF leakage from the closure site. The patient presented with a rare complication of pseudomeningocele at the site of his EVD incision. CT scan displayed dilation but not as significant as the prior enlarged ventricles from his IVH baseline. He underwent another EVD and later internalization with a ventriculoperitoneal shunt (VPS). The patient tolerated the procedures well with no further complications. Later, the thrombosis was resolved and Lovenox was no longer required. 

## Conclusions

There is no sole attributable cause to the development of CVST but rather it is multifactorial with additive effects that can manifest in unique ways. These symptoms include but are not limited to seizures, vomiting, hemiparesis, and the impact on cranial nerves. Due to the rapid and fatal consequences of this cerebrovascular event, prompt response with an antithrombotic must be administered to prevent any further complications. CVST is a rare phenomenon with an ambiguous clinical presentation creating a challenge for physicians to diagnose. This case gives insight into one of the many presentations in hopes of early detection and aggressive intervention of this condition. 
